# Volatile and non-volatile compound analysis of ginkgo chicken soup during cooking using a combi oven

**DOI:** 10.1016/j.fochx.2025.102276

**Published:** 2025-02-11

**Authors:** Lilan Chen, Jiale Huang, Can Yuan, Songcheng Zhan, Mingfeng Qiao, Yuwen Yi, Chunyou Luo, Ruixue Ma

**Affiliations:** a Sichuan Tourism University, Chengdu 610100, China; b College of Biomass Science and Engineering and Healthy Food Evaluation Research Center, Sichuan University, Chengdu, China; c Cuisine Science Key Laboratory of Sichuan Province, Sichuan Tourism University, Chengdu, China

**Keywords:** Combi oven, Ginkgo chicken soup, Aroma compounds, Taste compounds, Correlation analysis

## Abstract

This study employed a range of analytical techniques to evaluate the changes in both volatile and non-volatile compounds during different cooking times (30, 60, 90, 120, and 150 min) of ginkgo chicken soup prepared using a multifunctional combi oven, and comparedthese results with those obtained from the traditional ceramic pot method.The techniques included electronic nose (e-nose), electronic tongue (e-tongue), gas chromatography-ion mobility spectrometry (GC-IMS), high-performance liquid chromatography (HPLC), gas chromatography–mass spectrometry (GC–MS), and automated amino acid analysis. A total of 64 volatile compounds, primarily aldehydes, ketones, esters, and alcohols, were detected, with 23 key aroma components identified. Principal component analysis (PCA) demonstrated similar aroma and taste profiles between the two cooking methods. Additionally, 22 amino acids, 6 nucleotides enhancing umami, and 18 fatty acids were categorized into saturated, monounsaturated, and polyunsaturated groups. Pearson correlation revealed significant relationships among key amino acids, 5′-nucleotides, and volatile compounds, providing insights into industrial-scale applications of multifunctional ovens in ginkgo chicken soup production.

## Highlights

• Aroma and taste changes during gingko chicken soup cooking were studied.
• Flavors of combi oven-cooked and traditionally cooked chicken soups were compared.
• Both methods showed similar aroma and taste of the chicken soup samples.
• We identified the compounds contributing to flavor differences between samples.
• Our results support combi oven use for industrial ginkgo chicken soup production.

## 1 Introduction

Chicken soup is renowned for its nutritional value and distinctive taste. Ginkgo chicken soup is highly valued not only for its unique flavor but also for its health benefits, including stomach strengthening, lung nourishment, and blood pressure reduction, making it an essential component of chicken soup (Boateng & Yang, 2022; Li et al., 2022). Ginkgo chicken soup demonstrates broad industrial application potential, highlighting its versatility in various sectors. However, the traditional method of cooking ginkgo chicken soup in ceramic pots hinders large-scale production. Fortunately, with the growth of the catering industry, smart kitchen appliances designed specifically for broth cooking, including smart gas stoves, electric stewpots, and combi ovens, have rapidly gained popularity (Bi et al., 2021; Lai et al., 2022; Wang et al., 2024). For example, research has shown that electric stewpot can enhance the flavor of chicken soup by significantly increasing the content of FAAs and 5′-inosine monophosphate (IMP) (Zhang et al., 2018). In addition, another study highlighted the superior flavor-enhancing capabilities of combi ovens, which produce chicken soup with higher amino acid content and richer volatiles, offering better control over temperature and humidity, reducing cooking time, promoting healthier cooking, and ensuring stable food quality, all while outperforming smart gas stoves and electric stewpots in handling complex dishes (Wang et al., 2024). These findings underscore the potential of modern cooking appliances to enhance the flavor of traditional cuisines and serve as valuable resources for future research. Concurrently, it has been established that the stoves utilized in the preparation of chicken soup play a pivotal role in determining the soup's flavor profile.

Investigations into chicken soup extend beyond the development and refinement of culinary appliances, encompassing the effects of diverse elements on the taste profile. These elements include the choice of raw material types, ingredients, duration of the simmering process, and cooking temperature, all of which influence the formation of flavor compounds such as free amino acids, nucleotides, organic acids, and peptides (Duan et al., 2021; Qi et al., 2017; Qi et al., 2022). Despite notable progress in elucidating the quality and flavor nuances of chicken soup, there persists a scarcity of comprehensive studies delving into the implications of introducing supplementary ingredients, such as ginkgo nuts, on the holistic quality and taste profile of chicken soup. Ginkgo, as an essential ingredient in ginkgo chicken soup, significantly contributes to the dish's flavor profile through its inherent constituents. Ginkgo nuts are rich in terpenoid compounds, which impart a distinct nutty aroma and enhance the scent of baked goods and desserts with subtle notes of fruit or floral fragrances (Boateng & Yang, 2022; Liu et al., 2021). Currently, research that analyzes the flavor of traditional ginkgo chicken soup dishes cooked in a combi oven is lacking.

Therefore, we prepared ginkgo chicken soup using a combi oven to simulate the traditional production process of ceramic pot chicken soup. High-performance liquid chromatography (HPLC), gas chromatography-ion mobility spectrometry (GC-IMS), electronic nose (e-nose), electronic tongue (e-tongue), gas chromatography-MS (GC-MS), and automated amino acid analysis were employed to determine the volatile flavor- and taste-active compounds (free amino acids (FAAs), nucleotides, and fatty acids (FAs)) of chicken soup. We explored variations of aroma- and taste-contributing compounds during the cooking process and compared traditional ceramic pot and combi oven methodologies using multivariate statistical techniques. The purpose of this research was to ascertain the effect of combi ovens on the development of flavor in ginkgo chicken soup, providing a solid theoretical basis for modernized and convenient chicken soup production.

## 2 Materials and methods

### 2.1 Materials and reagents

The carcasses of Xiangjia hens were procured from Hunan Xiangjia Herding Co., Ltd., Hunan, China. Ingredients such as ginger, salt, green onions, and cooking wine were procured from a local Yonghui supermarket in Chengdu, China. White peppers were acquired from Chengdu Jingdong Huijia Trading Co., Ltd., Chengdu, China. Fresh ginkgo nuts were obtained from Kunming Liangzhi Trading Co. Ltd., based in Kunming, China. For our analytical work, 2-octanol of chromatographic purity and other analytical-grade chemical reagents were obtained from Sigma-Aldrich (Shanghai) Trading Co., Ltd., Shanghai, China. The 35-component FA mixture utilized in our assessments was also sourced from Sigma-Aldrich in Shanghai. High-grade solvents and reagents, including sulfosalicylic acid, petroleum ether, boron trifluoride, methanol, and hydrochloric acid, were secured from Chengdu Cologne Chemical Co., Ltd., Chengdu, China. Chemicals including ethanol, hexane, boron trifluoride, methanol, and sodium hydroxide used in our analyses were of the highest analytical grade and obtained from Shanghai Aladdin Biochemical Technology Co., Ltd., Shanghai, China.

### 2.2 Instruments and equipment

The following is a list of the instruments and equipment utilized in our study: a 20–2/1 iCombi Pro combi oven from Rational AG Co., based in Landsberg am Lech, Germany; FlavourSpec® GC-IMS, which is a product of the Aerospace Center in Cologne, Germany; the Fox 4000 electronic nose system and α-Astree electronic tongue system, both obtained from Toulouse Alpha MOS, Toulouse, France; the S 433 Amino Acid Analyzer from Sykam Ltd., Thedinghausen, Germany; the TRACE 1310 GC–MS, provided by Thermo Fisher Scientific, Waltham, MA, USA; and the API 3000 LC/MS/MS, sourced from SCIEX Co., CA, USA.

### 2.3 Sample preparation

Chicken soup was prepared following a modified version of our previous method (Wang et al., 2024). Prior to cooking, the chicken carcass was divided into two parts (excluding the head, neck, claws, wings, and visible fat), and thoroughly rinsed with water. Each half of chicken carcass (approximately 800 g) was cut into uniform pieces, approximately 3.0 ± 0.2 cm in size, and then placed into separate packaging bags for further use. The diced chicken was blanched for 1 min before being transferred to a cooking vessel. Five portions of chicken were cooked in a combi oven (designated CO-1 through CO-5), and one portion was cooked in a traditional ceramic pot (CP). For all samples, the chicken pieces, ginger slices, cooking wine, and water were combined in a weight ratio of 50:1:1:200.

To simulate the traditional cooking process of a ceramic pot, which involves initial high heat followed by low heat, the combi oven was set to steam-roast at 140 °C with 100 % humidity for 20 min. After this, foam was skimmed off, and the ginger and scallion were removed. Cooking continued at 100 °C for 30, 60, 90, 120, and 150 min, with samples taken at each time point. Samples were collected at 30, 60, and 90 min of simmering and designated as CO-1, CO-2, and CO-3, respectively. At the 90-min mark, pre-prepared ginkgo nuts were added to portions CO-4, CO-5, and CP, maintaining a meat-to-ginkgo nut ratio of 20:3. Ten minutes before the end of cooking, the soup was seasoned with salt and white pepper at a ratio of 200:1:0.1 (meat:salt:white pepper). Each chicken soup sample was prepared in triplicate.

After cooking, the soup underwent a lenitive filtration process to remove impurities and excess lipids. The broth was then allowed to cool to ambient temperature and filtered through food-grade degreasing cotton to achieve clarity, before being refrigerated for further analysis.

### 2.4 *E*-nose analysis

The chicken soup analysis was conducted using the FOX4000 electronic nose system (Toulouse Alpha MOS), which contains 18 metal oxide sensors (Wang et al., 2022; Wang et al., 2024). A sample of chicken broth (2.0 g) was placed into a 10.0 mL flask, heated to 50 °C, and the resulting vapor was directed into the sensor array at a flow rate of 150 mL/s for 300 s. The sensors, each with different functions, included the following: P40/1, T30/1, LY2/LG, LY2/AA, P10/2, T70/2, P30/1, LY2/gCT, LY2/Gh, LY2/gCT1, P10/1, PA/2, P40/2, P30/2, T40/2, T40/1, and TA/2 (Lv et al., 2024). These sensors were designed to detect a wide range of compounds, including alcohols, amines, sulfides, and fluorides, providing comprehensive chemical profiling. To ensure precision, each broth sample was tested five times. Data were analyzed to reduce complexity using principal component analysis (PCA) with Alphasoft software (version 2012.45). Duncan's test was applied to determine statistical significance, with a threshold set at *P* < 0.05.

### 2.5 GC-IMS analysis

Precisely 5.0 g of ginkgo chicken soup was weighed and placed in a 20 mL headspace vial for analysis, with three replicates per sample. The headspace sampling conditions were set as follows: incubation temperature of 50 °C, incubation time of 10 min, injection volume of 200 μL, incubation speed at 500 rpm, and an injector temperature of 85 °C. GC analysis was performed using an MXT-WAX column (30 m length × 0.53 mm internal diameter, 1 μm film thickness; Restek Co., Tokyo, Japan) at a column temperature of 60 °C, with high-purity nitrogen (≥99.99 %) as the carrier and drift gas. The total analysis time was 30 min, beginning with a flow rate of 2.0 mL/min held for 2 min, gradually increasing to 10 mL/min over the next 8 min, and then increasing to 100 mL/min between 10 and 20 min, held steady for the final 15 min. IMS conditions were maintained at 45 °C with a drift gas flow rate of 150 mL/min (N_2_ ≥ 99.99 %). Volatile organic compounds (VOCs) were identified by calculating their retention indices using 2-butanone (C_4_-C_8_) as an external standard, and these values, along with drift times, were compared with the GC-IMS library for qualitative analysis.

### 2.6 *E*-tongue analysis

In the electronic tongue experiment, this study selected the sixth sensor array from the ASTREE series. The system is equipped with an Ag/AgCl reference electrode and includes the following seven sensors: AHS-Sourceless, CTS-Saltiness, NMS-Umami, PKS, CPS, ANS, and SCS. Specifically, the AHS-Sourceless, CTS-Saltiness, and NMS-Umami sensors were utilized for the selective detection of sourness, saltiness, and umami, respectively (Zhang, Li, et al., 2024; Zhang, Liang, et al., 2024). For the analysis, the chicken soup was subjected to a two-stage filtration process using filter paper, followed by the transfer of 80 mL of the resulting filtrate into a 100 mL beaker. The testing protocol involved alternating exposure of chicken soup samples to equal volumes of deionized water for rinsing. Each sample underwent 10 trials, and the last five stable readings were averaged to derive the final results. The sensor responses were standardized to quantify the relative contribution of each taste parameter to ensure accurate and reproducible measurements (Liu et al., 2024).

### 2.7 Free amino acid (FAA) analysis

Samples were prepared as described by Li et al. (2024). Two milliliters of chicken soup was combined with 4 mL of a 3 % (*w*/*v*) sulfosalicylic acid solution, followed by centrifugation at 4 °C at 10,000 ×*g* for 15 min to isolate the supernatant. Subsequently, 2 mL of hexane solution was incorporated, and the solution was agitated to ensure thorough mixing. Thereafter, 2 mL of the aqueous phase was extracted, passed through a 0.22 μm filter, and the resulting filtrate was transferred into a sample container. The separation column measured 4.6 mm in inner diameter and 60 mm in length, with a set temperature of 57 °C. Detection wavelengths were 440 nm and 570 nm. The buffer flow rate was 0.4 mL/min, and the reaction solution containing ninhydrin had a flow rate of 0.35 mL/min. The sample injection volume was 20 μL, and the reaction unit was maintained at 140 °C. The experiment was performed in triplicate, each with three parallel samples, and the results are expressed as mean ± standard deviation.

### 2.8 Nucleotide analysis

Using a modified version of the method by Xu et al. (2021), 2 mL of each chicken soup sample was uniformly collected, diluted five-fold, filtered through a 0.22 μm membrane, and analyzed using HPLC. HPLC analyses utilized a SCIEX ChromXP C18 column (250 × 4.6 mm, 5 μm), compatible with SCIEX M3 MicroLC, MicroLC 200 Plus, and NanoLC 400 systems, with the column thermostat at 40 °C for peak resolution. The flow rate was maintained at 1.0 mL/min, detection wavelength at 260 nm, and injection volume at 10 μL. The mobile phase consisted of two solutions: solution A (20 mmol/L citric acid, 40 mmol/L triethylamine, and 0.1 % glacial acetic acid, pH 4.8) and solution B (methanol), with gradient elution. The prepared standard working solution or sample extract was injected into the HPLC system, and analysis was carried out under these parameters. Peak areas were recorded, ensuring that the response values fell within the instrument's linear detection range. Qualitative identification was determined by comparing retention times with standards, and quantification was performed using the external standard method (Zhan et al., 2020).

### 2.9 FA analysis

Adapting the procedure from Xiao et al. (2021), a measured sample was placed into a 100-mL colorimetric tube. For hydrolysis, 2 mL of 95 % ethanol, 4 mL of water, and 10 mL of 8.3 mol/L hydrochloric acid were added and mixed thoroughly. The mixture was then heated in an 80 °C water bath for 40 min, with periodic shaking to ensure uniformity. After cooling, 10 mL of 95 % ethanol was introduced, followed by extraction with a 100 mL mixture of ether and petroleum ether; this process was repeated thrice. The combined extracts were evaporated to dryness to recover the fat.

For fat saponification and FA methylation, 4 mL of a 2 % sodium hydroxide-methanol solution was added to the dried fat, heated at 45 °C for 20 min, followed by the addition of 4 mL of 14 % boron trifluoride-methanol; the mixture was heated again for another 20 min at the same temperature. After cooling, 3 mL of *n*-hexane was added, the mixture was shaken, and the upper layer was filtered through a 0.45-μm membrane.

A TG-FAME GC column (50 m × 0.25 mm, 0.20 μm) manufactured by Agilent Technologies was used for further analysis under the following conditions: initial temperature of 80 °C, ramping up to 160 °C at 20 °C/min, and increased to 230 °C at a rate of 5 °C/min. The injector was maintained at 260 °C, with a carrier gas flow rate of 0.63 mL/min and a split ratio of 100:1. MS analysis was conducted with an ion source temperature of 280 °C, a transfer line at 240 °C, a solvent delay of 4 min, and electron ionization set at 70 eV.

### 2.10 Statistical analysis

The experimental data were expressed as mean ± standard deviation using IBM SPSS software (Armonk, NY, USA) for statistical evaluation. PCA of e-nose and e-tongue data was directly conducted through the instrument's software; Origin 2021 software (OriginLab Co., Northampton, MA, USA) was employed for generating graphical representations. Additionally, orthogonal projections to latent structures discriminant analysis (OPLS-DA) and Pearson correlation analyses were performed using the SIMCA software (version 14.1; Sartorius, Göttingen, Germany).

## 3 Results and discussion

### 3.1 *E*-nose analysis

**Fig. 1:**
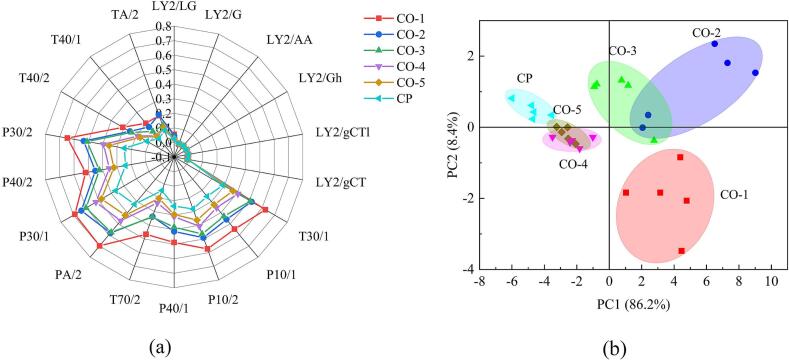
Electronic nose (*E*-nose) radar plot of chicken soup samples. CO, combi oven; CP, ceramic pot. (a) Radar map and (b) PCA of E-nose data.

Fig. 1a illustrates the radar plot of the e-nose analysis of chicken soup, and Table 1 lists the specific sensitive substances detected by each of the 18 sensors (Tian et al., 2023). We observed notable variations in the signal intensities of the T30/1, P10/1, P10/2, P40/1, T70/2, PA/2, P30/1, P40/2, P30/2, and T40/2 sensors. The signal intensity of these sensors followed the sequence: CO-1 > CO-2 > CO-3 > CO-4 > CO-5 > CP. The results indicated that the aroma concentration of CP-5 sample prepared using combi oven is stronger than that of CP sample prepared using ceramic pot. As observed in Table 1, the sensor is sensitive to polar compounds, hydrogen chloride, hydrocarbons, fluorine, alcohols, amines, ketones, chlorine, hydrogen sulfide, fluoride, and other substances, indicating that the chicken soup samples showed large differences in the above substances.

**Table 1:** Sensitive substance types for each sensor.

Serial Number	Sensor Name	Sensitive Substances
1	LY2/LG	Chlorine, Fluorine, Nitrogen Oxides, Sulfides
2	LY2/G	Ammonia, Amines, Nitrogen Oxides
3	LY2/AA	Ethanol, Acetone, Ammonia
4	LY2/Gh	Ammonia, Amines
5	LY2/gCTl	Sulfides
6	LY2/gCT	Propane, Butane
7	T30/1	Polar Compounds, Hydrogen Chloride
8	P10/1	Non-polar; Hydrocarbons, Ammonia, Chlorine
9	P10/2	Non-polar; Methane, Ethane
10	P40/1	Fluorine, Chlorine
11	T70/2	Toluene, Xylene, Carbon Monoxide
12	PA/2	Ethanol, Ammonia Solution, Amines
13	P30/1	Hydrocarbons, Ammonia, Ethanol
14	P40/2	Chlorine, Hydrogen Sulfide, Fluorides
15	P30/2	Hydrogen Sulfide, Ketones
16	T40/2	Chlorine
17	T40/1	Fluorine
18	TA/2	Ethanol

PCA employs dimensionality reduction to minimize the variable count within a data set, which streamlines model complexity and decreases computational intricacy (Leng et al., 2021). Fig. 1b depicts the PCA plot of chicken soup analyzed by e-nose; the horizontal axis represents first principal component (PC1), and the vertical axis represents second principal component (PC2). The combined individual contribution percentages of the two components, 86.2 % and 8.4 %, account for 94.6 % of the total, exceeding 85 % and thus indicating that the PCA results effectively capture the overall aroma profile of the six samples (Leng et al., 2021; Tian et al., 2023). In Fig. 1b, the data points for the six types of chicken soup samples are relatively clustered with overlapping regions, indicating that the e-nose did not significantly differentiate the chicken soup samples according to aroma. Additionally, the proportionate contribution of PC1 was considerably greater than that of PC2, indicating that the olfactory elements within the chicken soup samples showed more significant variations along the horizontal axis. The distance distributions of the other samples (CO-1, CO-2, and CO-3) were notably different. This suggests an evident difference in aroma between these three samples. The distribution of horizontal coordinate distances revealed similarities in the aroma profiles of CP, CO-4, and CO-5, indicating that the fragrance components of chicken soup prepared in a combi oven were similar to those of soup prepared in a traditional ceramic pot.

### 3.2 GC-IMS analysis

#### 3.2.1 GC-IMS flavor composition profiles in the ginkgo chicken soup during cooking

**Fig. 2:**
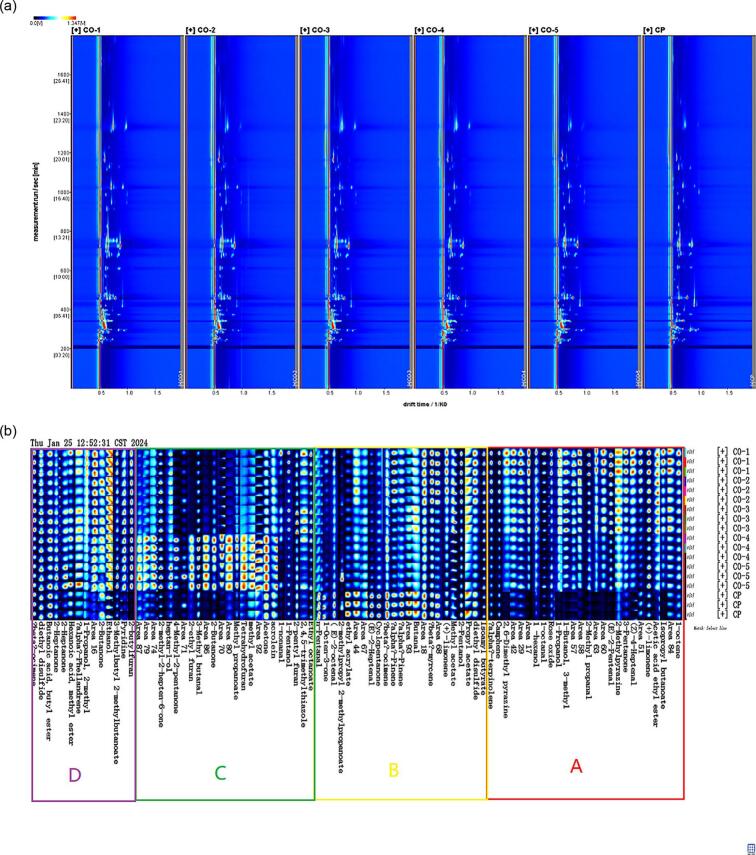
Gas chromatography–ion mobility spectrometry (GC–IMS) topographic and fingerprint chromatogram of volatile compounds in chicken soup samples. (a) Two-dimensional spectrum; (b) fingerprint chromatogram of volatile compounds. CO, combi oven; CP, ceramic pot.

Fig. 2a compares the 2D GC-IMS spectra of the ginkgo chicken soup cooked in a combi oven for various durations to that cooked in a traditional ceramic pot, arranged from left to right. The IMS ion drift duration is depicted on the horizontal axis, and the GC retention duration is indicated on the vertical axis. Distinctive red vertical lines within the 2D spectra mark reactive ion peaks, with dots on either side representing VOCs. The intensity of the color is indicative of its concentration, where redder and darker colors correspond to higher concentrations, whereas lighter colors correspond to lower concentrations (Chen et al., 2024; Fan et al., 2020; Guo et al., 2022). As shown in Fig. 2a, the uniform distribution of VOCs across the ginkgo chicken soup samples suggests the absence of notable variations in the VOC profiles detected. However, the color intensities of the reactive ion peaks showed significant differences among samples, indicating clear differences in VOC concentrations.

For an in-depth examination of VOC variability within diverse ginkgo chicken soup samples, the integrated NIST and IMS databases in the GC-IMS system were used for the qualitative identification of VOCs present in the samples by referencing retention indices, retention durations, and drift times. Fig. 2b presents a Gallery Plot (fingerprint) of VOCs from each ginkgo chicken soup sample, where the VOC concentration is directly proportional to the color intensity, with darker shades indicating higher concentrations (Guo et al., 2022; Wang et al., 2024). As shown in Fig. 2b, all chicken soup samples contained identical VOCs. In region A, CO samples contained compounds such as 1-octene, ethyl acetate, limonene, 2-methylpyrazine, 1-octanal, 1-hexanol, 2,6-dimethylpyrazine, and α-pinene, with these VOC concentrations gradually decreasing as cooking time increased. However, VOC concentrations in CP samples were lower than those in CO samples. In region B, all chicken soup samples contained the same types of VOCs, including propyl propionate, butyraldehyde, α-pinene, β-ocimene-M, cyclopentanone, (E)-2-heptenal, ethyl acrylate, 2-methylpropyl 2-methylpropanoate, (E)-2-octenal, 1-octen-3-one, and *n-*pentanal. The highest concentrations were observed in the CP sample. Moreover, VOC concentrations in the CO samples decreased with increasing cooking time. In region C, samples CO-4 and CO-5 exhibited higher levels of methyl acetate, butanone, ethyl furan, tetrahydrofuran, 3-methylbutanal, and 2-methyl-2-hepten-6-one, which may be attributed to the addition of gingko, the cooking time, and the cooking equipment used. In region D, the differences in VOCs such as 1-propanol, 2-butylfuran, α-phellandrene, 3-methylbutyl, pyridine, butanoic acid butyl ester, diethyl disulfide-D, and 2-methylbutanoate, were not pronounced among the CO samples.

#### 3.2.2 Qualitative GC-IMS analysis of volatile flavor compounds in the ginkgo chicken soup during cooking

**Fig. 3:**
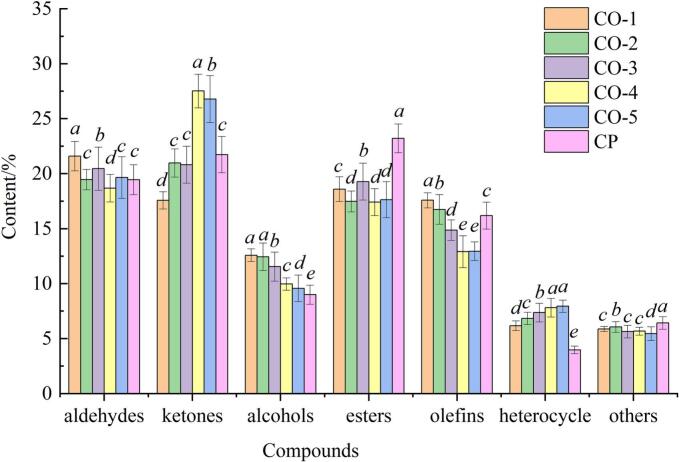
Relative contents of different compounds in chicken soup. CO, combi oven; CP, ceramic pot.

Qualitative assessment of aroma compounds in the ginkgo chicken soup samples was conducted by correlating the spectral signatures of the distinctive flavor constituents with the GC-IMS database entries (Wang et al., 2020). Table 2 shows that 64 distinct volatile flavor compounds were recognized and categorized into 11 aldehydes, 10 ketones, 8 alcohols, 13 esters, 11 olefins, 8 heterocyclic compounds, and 3 additional compounds belonging to other classes. Fig. 3 shows the relative contents of aromatic substances in the chicken soup samples.

**Table 2:** Volatile substances during the steaming process of ginkgo chicken soup samples.

Classes	Number	Compouds	CAS	Formula	RI^a^	RT/s^b^	DT/ms^c^
aldehydes	F1	(E)-2-octenal	C2548870	C_8_H_14_O	1441.3	1456.989	1.32862
	F2	(E)-2-Heptenal	C18829555	C_7_H_12_O	1330	1123.199	1.25591
	F3	1-octanal	C124130	C_8_H_16_O	1298.9	1030.065	1.81964
	F4	(Z)-4-Heptenal	C6728310	C_7_H_12_O	1213.8	754.156	1.61082
	F5	(E)-2-Pentenal	C1576870	C_5_H_8_O	1101.9	477.679	1.36015
	F6	3-Methyl butanal	C590863	C_5_H_10_O	923.9	301.755	1.40026
	F7	Butanal	C123728	C_4_H_8_O	886.4	280.211	1.11447
	F8	acrolein	C107028	C_3_H_4_O	838.8	252.833	1.05194
	F9	2-Methyl propanal	C78842	C_4_H_8_O	813.4	238.235	1.08857
	F10	n-Pentanal	C110623	C_5_H_10_O	998.3	346.048	1.17711
	F11	1-nonanal	C124196	C_9_H_18_O	1402.1	1339.378	1.93992
ketone	F12	2-methyl-2-hepten-6-one	C110930	C_8_H_14_O	1348.9	1179.893	1.17942
	F13	1-Octen-3-one	C4312996	C_8_H_14_O	1332.3	1130.135	1.6881
	F14	2-Heptanone	C110430	C_7_H_14_O	1190.6	680.146	1.26318
	F15	2-Heptanone	C110430	C_7_H_14_O	1189.2	676.9	1.62717
	F16	Cyclopentanone	C120923	C_5_H_8_O	1136.5	556.627	1.10753
	F17	3-Pentanone	C96220	C_5_H_10_O	1022.9	376.031	1.3433
	F18	4-Methyl-2-pentanone	C108101	C_6_H_12_O	989.2	339.276	1.48131
	F19	2-Butanone-D	C78933	C_4_H_8_O	913.4	295.7	1.24682
	F20	2-Butanone-M	C78933	C_4_H_8_O	913.7	295.88	1.05715
	F21	Acetone	C67641	C_3_H_6_O	853.3	261.189	1.11505
alcohol	F22	heptan-1-ol	C111706	C_7_H_16_O	1396.3	1322.011	1.40061
	F23	1 -hexanol	C111273	C_6_H_14_O	1365.8	1230.602	1.31726
	F24	1-Pentanol	C71410	C_5_H_12_O	1263.3	915.133	1.51272
	F25	1-Butanol,3-methyl	C123513	C_5_H_12_O	1214	754.805	1.48608
	F26	2-Pentanol	C6032297	C_5_H_12_O	1119.1	516.954	1.21402
	F27	1-Propanol, 2-methyl	C78831	C_4_H_10_O	1092.6	461.001	1.36015
	F28	1-Propanol	C71238	C_3_H_8_O	1042.7	400.151	1.1133
	F29	Ethanol	C64175	C_2_H_6_O	941.7	311.979	1.12795
Esters	F30	3-Methylbutyl,2-methylbutanoate	C27625350	C_10_H_20_O_2_	1276.4	957.737	1.42503
	F31	Hexanoic acid, methyl ester	C106707	C_7_H_14_O_2_	1192.8	685.916	1.6909
	F32	Isopropyl butanoate	C638119	C_7_H_14_O_2_	1042.1	399.386	1.2513
	F33	Acetic acid ethyl ester	C141786	C_4_H_8_O_2_	896.5	285.974	1.09154
	F34	Propyl acetate	C109604	C_5_H_10_O_2_	922.8	301.104	1.16658
	F35	Methyl acetate-D	C79209	C_3_H_6_O_2_	845.7	256.786	1.20372
	F36	Methyl propanoate	C554121	C_4_H_8_O_2_	917.6	298.11	1.32563
	F37	methyl acetate-M	C79209	C_3_H_6_O_2_	887.8	280.996	1.19455
	F38	Ethyl octanoate	C106321	C_10_H_20_O_2_	1403.5	1343.713	1.47671
	F39	Isoamyl butyrate	C106274	C_9_H_18_O_2_	1302.9	1041.905	1.4018
	F40	Butanoic acid, butyl ester	C109217	C_8_H_16_O_2_	1192.4	684.611	1.33053
	F41	ethyl acrylate	C140885	C_5_H_8_O_2_	996.5	343.827	1.42447
	F42	2-methylpropyl 2-methylpropanoate	C97858	C_8_H_16_O_2_	1067.4	430.226	1.80441
alkenes	F43	α-terpinolene	C586629	C_10_H_16_	1285.8	988.452	1.21833
	F44	β-ocimene	C13877913	C_10_H_16_	1263.3	915.133	1.25591
	F45	β-ocimene	C13877913	C_10_H_16_	1206.7	730.974	1.21438
	F46	(+)-limonene	C138863	C_10_H_16_	1197	699.622	1.21615
	F47	(+)-limonene	C138863	C_10_H_16_	1205.6	727.538	1.72124
	F48	Camphene	C79925	C_10_H_16_	1063.4	425.421	1.21188
	F49	α-Pinene-M	C80568	C_10_H_16_	1022.9	376.031	1.29511
	F50	α-Pinene-D	C80568	C_10_H_16_	1023.2	376.414	1.66531
	F51	1-octene	C111660	C_8_H_16_	831.7	248.774	1.45555
	F52	β-myrcene	C123353	C_10_H_16_	1160.8	611.922	1.21836
heterocycles	F53	2,4,5-trimethylthiazole	C13623115	C_6_H_9_NS	1403.4	1343.369	1.55974
	F54	2,6-Dimethyl pyrazine	C108509	C_6_H_8_N_2_	1355.1	1198.661	1.14002
	F55	2-pentyl furan	C3777693	C_9_H_14_O	1238.7	835.179	1.23743
	F56	Pyridine	C110861	C_5_H_5_N	1215.9	761.139	1.24241
	F57	2-Methylpyrazine	C109080	C_5_H_6_N_2_	1213.4	752.857	1.39405
	F58	2-butylfuran	C4466244	C_8_H_12_O	1101.7	477.141	1.17622
	F59	2-ethyl furan	C3208160	C_6_H_8_O	961.9	323.578	1.28854
	F60	Tetrahydrofuran	C109999	C_4_H_8_O	894.8	285.019	1.22221
Others	F61	Rose oxide	C16409431	C_10_H_18_O	1333.6	1134.098	1.34673
	F62	diethyl disulfide	C110816	C_4_H_10_S_2_	1214.4	756.104	1.29385
	F63	diethyl disulfide	C110816	C_4_H_10_S_2_	1161.5	613.588	1.28769

a Retention index; ^b^ Retention time; ^c^ Draft tome.

The aldehyde content accounted for 18.68–21.6 % of the total volatile flavor compounds, and little variation was observed in aldehyde levels among the six samples. Primarily, aldehydes are derived from lipids that undergo oxidative and degradative processes. Because of their low threshold values and relatively high concentrations, aldehydes are crucial aroma compounds in ginkgo chicken soup, contributing to its rich, fatty, and meaty aroma (Qi et al., 2023; Tanimoto et al., 2015). Previous studies have indicated that (E)-2-heptenal, 1-octanal, and 1-nonanal are the key odorous compounds in ginkgo chicken soup (Feng et al., 2018; Wang et al., 2016). Octanal and nonanal compounds are produced by the oxidation of oleic acid (Chen et al., 2021). Based on the FA analysis, which indicates a notably high concentration of oleic acid in ginkgo chicken soup, it is likely that oleic acid plays a substantial role in shaping the soup's aroma profile. Furthermore, the FA content was consistent with the results of the volatile compound analysis.

The ketone content ranged from 17.58 % to 27.53 % and primarily originated from the oxidation and decomposition of fats, as well as the metabolic processes of carbohydrates. Ketones typically exhibit floral, fruity, and creamy aromas. However, due to their relatively high detection thresholds, these compounds have a minimal impact on the overall flavor profile of ginkgo chicken soup, and their contribution remains subtle compared to more dominant flavor compounds (Zhang et al., 2022). Notably, CO-4 and CO-5, the ketone content was significantly higher, with notable increases in 2-methyl-2-hepten-6-one, 4-methyl-2-pentanone, and acetone compared to other samples. This observation may be attributed to the addition of gingko, cooking time, and cooking equipment.

Alcohol content ranged from 9.00 % to 12.58 %. The presence of these compounds is predominantly attributed to the oxidative processes involving unsaturated FAs. Owing to their relatively high odor thresholds, alcohols play a minor role in shaping the overall flavor of ginkgo chicken soup. However, they still impart distinct and pleasant aromas, such as floral and fruity notes (Ge et al., 2023). As the cooking time increased, the alcohol content decreased, which may be attributed to their conversion into aldehydes and other substances (Lai et al., 2022). The CP samples showed a lower content of alcohol compounds than those cooked in the combi oven. The substances identified in ginkgo chicken soup, such as 1-hexanol and 1-pentanol, are primarily produced by the oxidation of linoleic acid, resulting in a fresh and fragrant aroma that enhances the overall fragrance of the soup (Meng et al., 2022). Given the relatively high concentration of linoleic acid in the samples, it is possible that 1-hexanol and 1-pentanol contributed to the soup's aroma.

The ester concentration ranged from 17.41 % to 23.21 % and was primarily derived from the esterification reaction between alcohols and organic acids, which typically exhibit a fruity aroma. Sample CP contained a relatively high level of alcohol, accounting for 23.21 % of all volatile flavor compounds, suggesting that esters are likely to contribute to the equilibrium of the overall taste profile in ginkgo chicken soup (Zhou et al., 2022). Olefins constituted 12.91–17.58 % of the total volatile flavor content, with lower levels found in samples CO-4 and CO-5. Owing to their low threshold values, olefins showed a less significant contribution to flavor formation. Heterocyclic compounds represented 3.98–7.94 % of all volatile flavor compounds, with furan being a crucial heterocyclic compound in meat flavors. Furan, characterized by a low olfactory threshold and a vegetable-like scent (Andrés et al., 2002), significantly impacts the flavor profile of ginkgo chicken soup. Samples CO-4 and CO-5 were rich in tetrahydrofuran and 2-ethylfuran, whereas 2-butylfuran was present at lower concentrations in sample CP. Other compounds accounted for 5.45–6.42 % of all volatile flavor compounds, and diethyl disulfide was detected in all samples; this compound enhances the overall flavor and aroma of meat (Zhang, Li, et al., 2024; Zhang, Liang, et al., 2024). In summary, compared to the CP sample prepared in a ceramic pot, the CO-5 sample showed higher levels of aldehydes, ketones, alcohols, and heterocyclic compounds.

### 3.3 *E*-tongue analysis

**Fig. 4:**
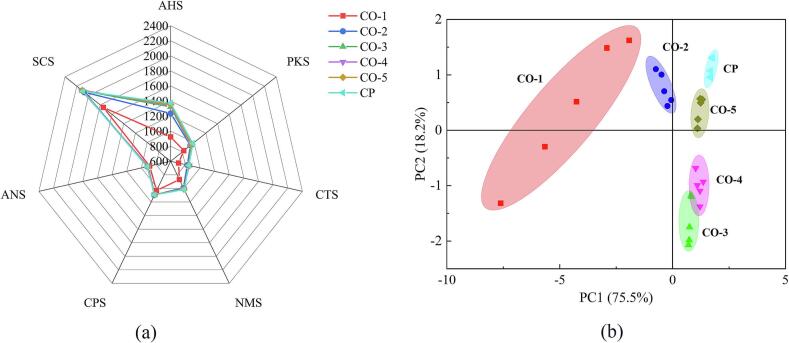
Electronic tongue (E-tongue) radar plot of chicken soup samples. CO, combi oven; CP, ceramic pot. (a) Radar map and (b) PCA of E-tongue data.

Fig. 4a displays the flavor radar plot generated by e-tongue analysis of different ginkgo chicken soup samples. With the exception of sample CO-1, the relative response intensities of the seven sensors for the remaining samples (CO-2, CO-3, CO-4, CO-5, and CP) did not differ significantly. Notably, the relative response intensities of the acidic and umami sensors showed the following order: CP > CO-5 > CO-4 > CO-3 > CO-2 > CO-1. For saltiness sensors, the relative response intensities showed the following order: CO-4 > CO-3 > CP > CO-5 > CO-2 > CO-1. Among the various taste components, umami is predominantly from the proteins and amino acids found within the soup's ingredients. As the soup simmers, proteins and amino acids from the chicken slowly release into the liquid, undergoing several chemical reactions. These include lipid oxidation and the Maillard reaction, both of which are facilitated by heat and enhance the soup's umami flavor. Additionally, non-volatile compounds such as FAAs and nucleotides play a significant role in the perception of umami. Acidity mainly comes from organic acids and FAAs, while saltiness primarily results from salts like sodium chloride (Shakoor et al., 2022).

Fig. 4b presents the PCA plots of the different chicken soup samples analyzed using an e-tongue. The PCA findings suggests that PCA effectively differentiated the ginkgo chicken soup samples based on varying cooking durations. According to Fig. 4b, the differences among the ginkgo chicken soup samples were more prominent in PC1 compared to PC2. The data points representing the ginkgo chicken soup samples were fairly clustered, mainly distributed across the first, second, and fourth quadrants. Samples CO-3 and CO-4 were both positioned in the fourth quadrant with partial overlap, indicating minimal differences between these samples. In contrast, ginkgo chicken soup samples CO-1, CO-2, and CO-5 showed notable differences. Furthermore, the ginkgo chicken soup samples CO-5 and CP positioned in the second quadrant displayed comparable horizontal coordinate distribution distances. This suggests that the flavor profiles of ginkgo chicken soup made in a combi oven are similar to those cooked in a traditional ceramic pot.

### 3.4 Analysis of FAAs and 5′-nucleotides

Table 3 shows the content and taste-active value (TAV) of FAAs and 5′-nucleotides in ginkgo chicken soup samples. The FAAs were categorized into four principal categories based on their taste profiles: glutamic acid and aspartic acid (umami); alanine, glycine, proline, serine, and threonine (sweet); arginine, histidine, isoleucine, leucine, lysine, phenylalanine, tyrosine, and valine (bitter); and methionine along with cysteine (lacking distinctive taste) (Xu et al., 2021). Through the detection of 5′-nucleotides, identified hypoxanthine, inosine monophosphate (IMP), adenosine diphosphate, inosine, and adenosine monophosphate, whereas guanosine monophosphate was not detected in any samples. Chicken soup samples exhibited high mass concentrations of nucleotides, such as hypoxanthine, IMP, and adenosine diphosphate, which typically impart a bitter and slightly medicinal taste, an umami taste, and no flavor, respectively. Notably, CO-5, the hypoxanthine concentration was significantly low, which may be related to the heating time and method.

**Table 3:** Composition of nucleotides and free amino acids in ginkgo chicken soup samples.

taste	Number	FAA name	Threshold mg/10 mL	Free Amino Acid content(mg/10 mL)						TAV					
				CO-1	CO-2	CO-3	CO-4	CO-5	CP	CO-1	CO-2	CO-3	CO-4	CO-5	CP
umami	FAA1	ASP	10	3.85 ± 0.41^d^	4.11 ± 0.27^c^	4.36 ± 0.43^b^	4.67 ± 0.34^a^	4.72 ± 0.33^c^	4.45 ± 0.21^b^	0.39	0.41	0.44	0.47	0.47	0.45
	FAA2	GLU	3	5.86 ± 0.62^d^	6.58 ± 0.74^c^	6.64 ± 0.48^c^	6.92 ± 0.82^b^	7.32 ± 0.49^b^	8.83 ± 0.65^a^	1.95	2.19	2.21	2.31	2.44	2.94
		Total		9.71	10.69	11.00	11.59	12.04	13.28						
sweet	FAA3	THR	26	1.56 ± 0.25^d^	1.66 ± 0.13^c^	1.74 ± 0.08^b^	1.76 ± 0.22^b^	1.82 ± 0.15^a^	1.85 ± 0.22^a^	0.06	0.06	0.07	0.07	0.07	0.07
	FAA4	SER	15	2.55 ± 0.08^c^	2.63 ± 0.21^b^	2.78 ± 0.14^a^	2.86 ± 0.12^c^	3.11 ± 0.17^b^	3.04 ± 0.33^a^	0.17	0.18	0.19	0.19	0.21	0.20
	FAA5	GLY	13	1.95 ± 0.08^d^	2.05 ± 0.07^c^	2.09 ± 0.22^c^	2.13 ± 0.17^c^	2.25 ± 0.14^b^	2.34 ± 0.15^a^	0.15	0.16	0.16	0.16	0.17	0.18
	FAA6	PRO	30	1.01 ± 0.08^d^	1.06 ± 0.02^c^	1.12 ± 0.07^b^	1.19 ± 0.14^b^	1.31 ± 0.06^b^	1.38 ± 0.23^a^	0.03	0.04	0.04	0.04	0.04	0.05
	FAA7	ALA	6	2.95 ± 0.18^f^	3.16 ± 0.49^e^	3.28 ± 0.23^d^	3.41 ± 0.17^c^	3.87 ± 0.12^b^	4.03 ± 0.15^a^	0.49	0.53	0.55	0.57	0.65	0.67
		Total		10.02	10.56	10.99	11.35	12.36	12.64						
bitter	FAA8	ILE	9	0.89 ± 0.05^e^	1.05 ± 0.06^d^	1.11 ± 0.12^c^	1.17 ± 0.05^b^	1.23 ± 0.14^a^	1.21 ± 0.14^a^	0.10	0.12	0.12	0.13	0.14	0.13
	FAA9	LEU	19	1.57 ± 0.06^d^	1.76 ± 0.11^c^	1.84 ± 0.08^c^	1.96 ± 0.14^b^	2.13 ± 0.07^a^	2.16 ± 0.24^a^	0.08	0.09	0.10	0.10	0.11	0.11
	FAA10	VAL	4	1.38 ± 0.06^d^	1.69 ± 0.07^c^	1.71 ± 0.14^b^	1.79 ± 0.12^b^	1.83 ± 0.09^b^	1.88 ± 0.34^a^	0.39	0.44	0.46	0.49	0.53	0.54
	FAA11	TYR		0.91 ± 0.03^d^	1.11 ± 0.12^c^	1.17 ± 0.11^c^	1.19 ± 0.06^c^	1.25 ± 0.14^b^	1.36 ± 0.17^c^						
	FAA12	PHE	9	1.15 ± 0.06^e^	1.32 ± 0.12^d^	1.39 ± 0.03^c^	1.44 ± 0.05^b^	1.56 ± 0.07^a^	1.63 ± 0.15^a^	0.13	0.15	0.15	0.16	0.17	0.18
	FAA13	HIS	2	7.21 ± 0.21^d^	7.39 ± 0.32^c^	7.73 ± 0.27^b^	7.89 ± 0.19^b^	8.09 ± 0.26^b^	8.65 ± 0.45^a^	3.61	3.70	3.87	3.95	4.05	4.33
	FAA14	LYS	5	2.14 ± 0.06^a^	2.44 ± 0.11^a^	2.17 ± 0.14^a^	1.39 ± 0.05^d^	1.75 ± 0.07^c^	2.01 ± 0.12^a^	0.43	0.49	0.43	0.28	0.35	0.40
	FAA15	ARG	5	0.75 ± 0.03^b^	1.63 ± 0.05^a^	1.72 ± 0.08^a^	1.95 ± 0.06^a^	0.48 ± 0.03^c^	0.33 ± 0.07^c^	0.15	0.33	0.34	0.39	0.10	0.07
		Total		15.00	15.39	18.84	18.78	18.32	19.23						
tasteless	FAA16	CYS		0.12 ± 0.02^b^	0.13 ± 0.03^b^	0.15 ± 0.07^a^	0.16 ± 0.06^a^	0.17 ± 0.04^a^	0.18 ± 0.06^a^						
	FAA17	MET		0.56 ± 0.02^d^	0.71 ± 0.03^c^	0.84 ± 0.08^b^	0.90 ± 0.11^a^	0.95 ± 0.05^a^	0.91 ± 0.08^a^						
		Total		0.68	0.84	0.84	1.06	1.12	1.09						
5′-nucleotide	N1	HX	–	9.88 ± 0.45^b^	10.85 ± 0.57^a^	10.51 ± 0.40^a^	11.38 ± 0.41^a^	6.35 ± 0.34^d^	8.07 ± 0.36^c^						
	N2	IMP	2.5	5.41 ± 0.34^b^	3.94 ± 0.17^c^	6.58 ± 0.37^a^	2.42 ± 0.11^d^	5.81 ± 0.27^b^	7.44 ± 0.32^a^	2.16	1.58	2.63	0.97	2.32	2.98
	N3	ADP	–	9.25 ± 0.59^bc^	9.69 ± 0.32^b^	12.74 ± 0.58^a^	8.48 ± 0.48^c^	8.81 ± 0.38^c^	9.51 ± 0.34^b^						
	N4	HXR	–	0.87 ± 0.11^e^	1.35 ± 0.06^c^	0.73 ± 0.09^e^	1.33 ± 0.05^cd^	1.59 ± 0.09^b^	2.08 ± 0.10^a^						
	N5	GMP		–	–	–	–	–	–						
	N6	AMP	5.0	1.03 ± 0.05^d^	1.31 ± 0.08^bc^	0.98 ± 0.12^d^	1.38 ± 0.06^b^	1.06 ± 0.06^cd^	1.54 ± 0.06^a^	0.21	0.26	0.20	0.28	0.21	0.31
		TN		26.44	26.14	31.54	24.99	23.62	28.64						
		TFAA		35.41	37.48	41.67	42.78	43.84	46.24						

FAA: free amino acid, TN: total nucleotides, TFAA:total free amino acids, TAV:taste-active value. Note: Different lowercase letters indicate significant differences between samples.

Table 3 illustrates that the total free amino acids (TFAA) concentrations in ginkgo chicken soup samples, which were prepared with extended cooking times, correspond to the following sequence: 35.41, 37.48, 41.69, 42.78, 43.84, and 46.24 mg per 100 mg of sample for CO-1, CO-2, CO-3, CO-4, CO-5, and CP, respectively. During the soup preparation process, an extended cooking period allows for the heat to promote the migration of FAAs from the chicken into the broth. Consequently, this results in a rise in the overall concentration of FAAs (Kong et al., 2017). In the ginkgo chicken soup samples, the ranking of umami and sweet amino acid levels was as follows: CP was the highest, followed by CO-5, CO-4, CO-3, CO-2, and CO-1 in descending order. In contrast, the sequence for bitter amino acids was CP, CO-3, CO-4, CO-2, CO-5, and CO-1. Among the three classes of taste-active amino acids identified, bitter amino acids were found to be the most prevalent, followed by sweet and umami. Notably, despite their high concentration, bitter amino acids are not taste-active (Chen et al., 2019). The TAV was used to evaluate key flavor-active amino acids. A TAV value exceeding 1 signifies that the particular flavor compound is a significant taste determinant in ginkgo chicken soup; conversely, a TAV value <1 implies that the amino acid exerts a modulatory influence on the soup's taste profile (Kong et al., 2017). As shown in Table 3, histidine and glutamic acid had TAV > 1, whereas all other amino acids showed TAV < 1. Therefore, histidine and glutamic acid contributed significantly to the taste of ginkgo chicken soups. Nevertheless, given that bitter amino acids lack taste activity, the predominant flavor characteristic of the soup samples was umami. In addition, the TAV value of 5′-IMP was greater than one. The IMP content, with a known threshold value, tended to increase gradually with increasing cooking time, reaching a peak in sample CO-3. The higher IMP content in sample CO-5 than in sample CO-4 may be attributed to the greater leaching of nutrients from gingko nuts as the heating time increased. In comparison to sample CP, sample CO-5 exhibited reduced concentrations of total nucleotides, free amino acids, umami and sweet amino acids.

### 3.5 FA analysis

FAs can undergo oxidation and degradation to produce aldehydes, alcohols, carboxylic acids, and other compounds, which generate a pleasant meaty aroma and affect the smell of ginkgo chicken soup (Jia et al., 2024). In the analysis of the ginkgo chicken soup samples, a total of 18 FAs were detected, as detailed in Table 4. This included 7 monounsaturated FAs (MUFAs), 6 saturated FAs (SFAs), and 5 polyunsaturated FAs (PUFAs). As for FA content in the samples, MUFAs were the most abundant followed by SFAs and then PUFAs. The concentrations of MUFAs and PUFAs decreased with increasing cooking time, whereas those of SFAs increased with increasing cooking time. During cooking, unsaturated FAs (MUFAs and PUFAs) are more prone to oxidation than SFAs, decomposing into volatile flavor compounds such as octanal and nonanal (Shakoor et al., 2022). As indicated in Table 4, the ginkgo chicken soup samples had higher MUFA content than PUFA content. The prevalent monounsaturated FAs identified were oleic acid (C18: 1n9c), palmitoleic acid (C16:1), eicosenoic acid (C20:1), and transvaccenic acid (C18:1n9t), which are presumed to have made a substantial contribution to the aromatic profile of the ginkgo chicken soups. Sample CO-5 exhibited reduced concentrations of both MUFAs and PUFAs relative to sample CP.

**Table 4:** Fatty acid content of ginkgo chicken soup samples.

	Sample/μg/㎏					
	CO-1	CO-2	CO-3	CO-4	CO-5	CP
C16:0	723.34 ± 30.68^e^	745.34 ± 92.68^d^	698.25 ± 81.67^c^	761.55 ± 48.71^b^	775.34 ± 52.28^a^	782.35 ± 23.56^a^
C17:0	6.35 ± 0.97^d^	7.49 ± 1.25^b^	8.55 ± 1.59^a^	7.33 ± 1.12^b^	6.25 ± 2.18^d^	6.52 ± 1.25^c^
C18:0	425.96 ± 35.93^d^	476.46 ± 65.74^d^	527.23 ± 29.68^c^	595.13 ± 19.25^b^	636.59 ± 33.17^a^	668.31 ± 45.57^a^
C19:0	43.27 ± 9.43^b^	45.63 ± 13.51^b^	48.51 ± 7.34^b^	52.34 ± 8.03^b^	61.52 ± 1.25^a^	63.18 + 10.35^a^
C20:0	25.76 ± 6.37^d^	22.92 ± 5.48^d^	28.56 ± 2.45^c^	35.39 ± 1.73^b^	42.44 ± 4.38^a^	44.13 ± 4.35^a^
C22:0	48.72 ± 1.12^a^	54.23 ± 1.24^a^	60.83 ± 1.33^b^	71.24 ± 1.97^a^	75.41 ± 1.34^a^	74.32 ± 1.66^a^
SFAs	1273.40	1351.07	1371.93	1522.98	1597.55	1638.81
C14:1	6.45 ± 1.32^d^	8.38 ± 2.13^b^	10.16 ± 2.65^a^	7.59 ± 0.83^c^	6.87 ± 1.54^d^	9.66 ± 1.51^ab^
C16:1	156.57 ± 29.37^d^	183.11 ± 21.52^b^	208.93 ± 18.48^a^	177.82 ± 26.14^c^	141.22 ± 14.78^e^	155.67 ± 16.53^d^
C17:1	9.53 ± 2.36^e^	9.35 ± 1.11^e^	10.48 ± 2.93^d^	12.71 ± 0.66^c^	14.21 ± 1.23^d^	16.17 ± 1.36^a^
C18:1n9t	693.17 ± 24.85^a^	673.32 ± 58.86^b^	656.76 ± 16.69^c^	642.70 ± 86.81^b^	616.98 ± 58.55^d^	627.33 ± 57.88^c^
C18:1n9c	697.25 ± 64.74^a^	681.62 ± 62.73^b^	654.48 ± 96.52^c^	637.48 ± 82.08^d^	625.24 ± 38.98^e^	618.46 ± 39.32^e^
C20:1	89.45 ± 6.13^c^	75.93 ± 8.98^a^	72.37 ± 8.60^b^	68.67 ± 4.11^c^	53.59 ± 4.69^d^	54.86 ± 6.47^d^
C22;1n9	11.72 ± 1.14^b^	13.89 ± 0.45^a^	9.86 ± 1.08^c^	8.93 ± 0.56^d^	6.93 ± 1.32^e^	7.29 ± 0.81^e^
MUFAs	1663.14	1645.60	1622.04	1555.90	1464.04	1489.44
C18;2n6	684.17 ± 36.27^a^	658.92 ± 55.43^b^	613.27 ± 48.23^c^	615.64 ± 36.14^b^	602.53 ± 42.32^c^	593.85 ± 47.73^d^
C20:2	49.92 ± 4.16^a^	45.51 ± 0.89^b^	35.35 ± 2.23^c^	24.31 ± 2.52^d^	22.08 ± 1.75^e^	26.67 ± 2.34^e^
C20:3n6	20.53 + 1.12^e^	33.83 ± 3.35^b^	40.43 ± 1.35^a^	28.56 ± 1.41^c^	26.16 ± 2.78^c^	24.27 ± 2.29^d^
C20:3n3	2.12 ± 0.53^b^	2.54 ± 0.23^d^	2.92 ± 0.38^bc^	2.84 ± 0.31^c^	3.12 ± 0.52^a^	3.09 ± 0.44^a^
PUFAs	756.74	740.80	691.97	671.35	653.89	647.88

MUFAs: monounsaturated fatty acids, PUFAs: polyunsaturated fatty acids, SFAs: saturated fatty acids.

### 3.6 OPLS-DA analysis of ginkgo chicken soup samples

**Fig. 5:**
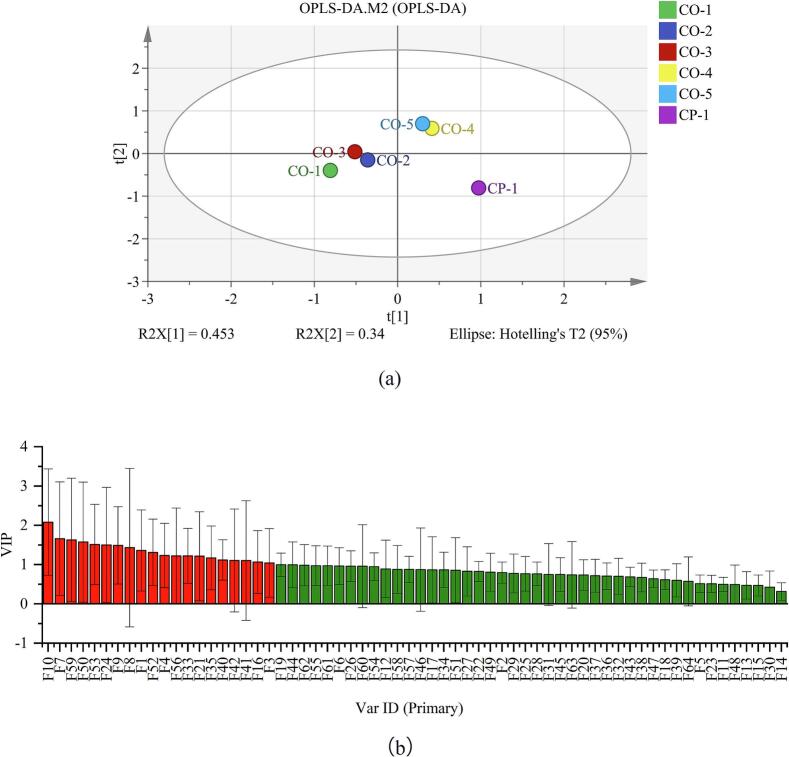
OPLS-DA analysis of aroma compounds in chicken soup samples: (a) OPLS-DA; (b) variable importance in projection (VIP).

**Fig. 6:**
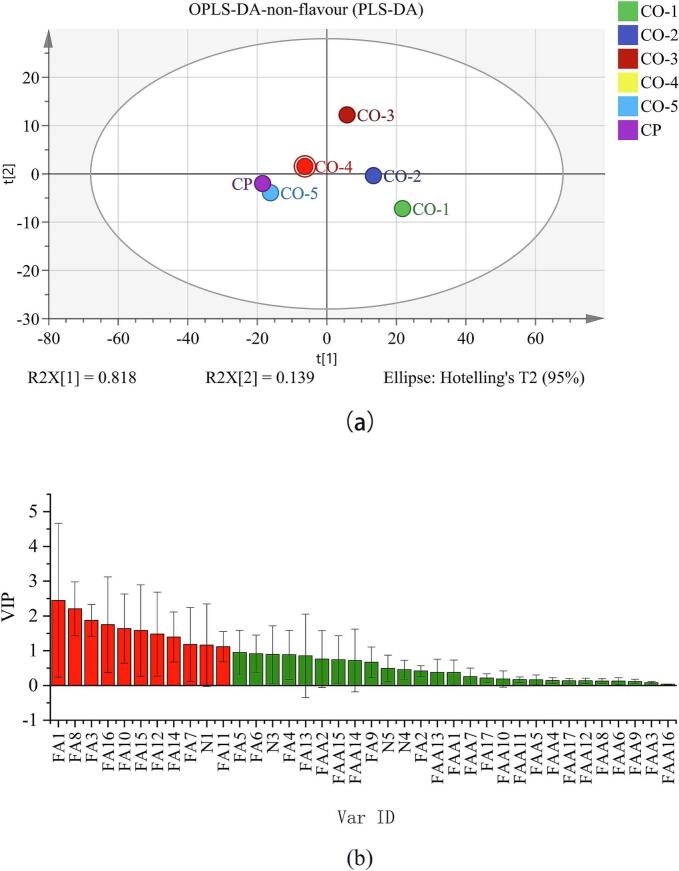
OPLS-DA analysis of non-volatile compounds in chicken soup samples: (a) OPLS-DA; (b). variable importance in projection (VIP).

The OPLS-DA technique is an effective multivariate data analysis approach particularly well-suited to analyzing and interpreting patterns and relationships within complex datasets. Its strengths lie in effectively distinguishing different sample groups, offering intuitive data interpretation, and maintaining robustness even in the presence of noise and multicollinearity (Zhang, Li, et al., 2024; Zhang, Liang, et al., 2024). To elucidate the distinctions between volatile and non-volatile constituents across diverse ginkgo chicken soup samples, the data pertaining to these compounds from each sample were subjected to OPLS-DA pattern recognition for analysis (Fig. 5, Fig. 6).

Fig. 5a illustrates that ginkgo chicken soup samples were well separated in the OPLS-DA score plot. Samples CO-1, CO-2, and CO-3 were clustered together, indicating similar aromatic characteristics. Additionally, CO-4 and CO-5 were close to each other, suggesting similar aromatic profiles. Notably, the CP sample, positioned in the fourth quadrant and distant from the CO series samples, demonstrated a clear differentiation in aroma profiles attributable to the distinct cooking techniques. Fig. 5b displays the variable importance in projection (VIP) scores of ginkgo chicken soup samples, which reflect the significance of variables during the cooking process. The VIP score is indicative of the relative influence exerted by different flavor compounds, where a higher score denotes a more substantial contribution (Xu et al., 2021). The compounds with VIP scores >1 were 2-methyl-2-hepten-6-one, acetone, 2-methylpropanal, 4-methyl-2-pentanone, 2-butanone, 2-pentylfuran, 2-methylpropyl 2-methylpropanoate, ethyl acrylate, ethyl octanoate, 3-methylbutanal, nonanal, propyl propionate, (+)-limonene, 3-methylbutyl 2-methylbutanoate, methyl hexanoate, 2-pentanol, (E)-2-pentenal, 2-butylfuran, (E)-2-heptenal, pentyl butyrate, 1-octanal, camphene, methyl formate, and 1-octene. These compounds include 6 aldehydes, 4 ketones, 1 alcohol, 8 esters, 3 alkenes, and 2 heterocyclic compounds.

According to the OPLS-DA pattern recognition analysis of non-volatile compound data (Fig. 6a), CP and CO-5 were clustered together, indicating similar flavor characteristics among these samples, whereas CO-1, CO-2, CO-3, and CO-4 were relatively dispersed, indicating significant differences in flavor among these samples. Compounds with VIP scores greater than 1 included hypoxanthine, tyrosine, C18:1n9t, C16:1, C20:2, C18:0, C16:0, C14:1, C20:1, C18:2n6., and C20:3n6 (Fig. 6b), indicating that these compounds were the primary contributors to differences in taste.

### 3.7 Correlation analysis

**Fig. 7:**
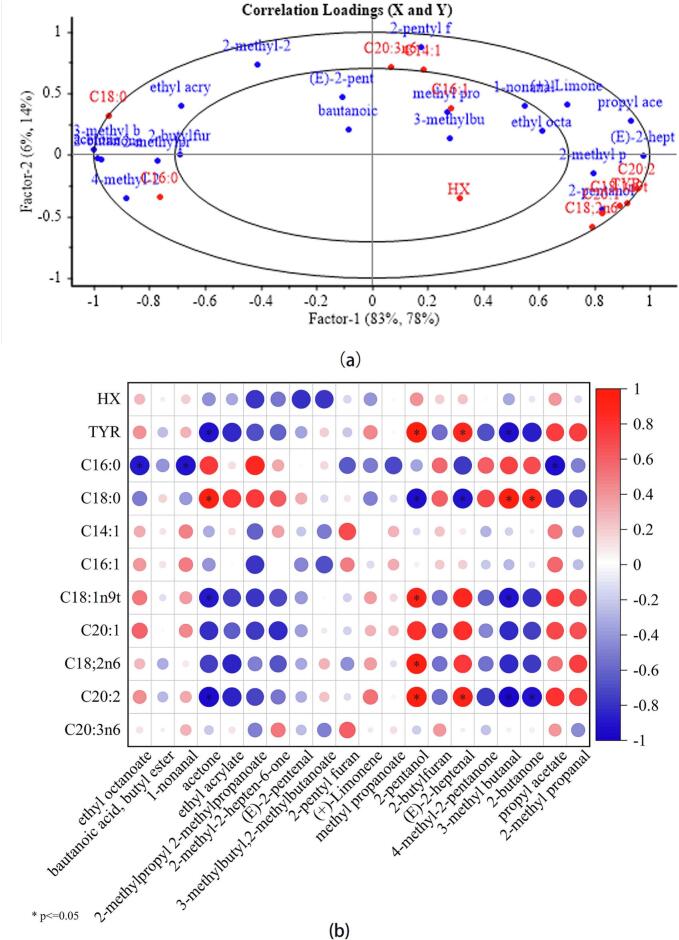
Correlation analysis of key aroma compounds (volatile compounds) and aroma attributes (non-volatile compounds): (a) partial least squares regression; (b) Pearson correlation. Red and blue signify affirmative (0 < *r* < 1) and adverse (−1 < *r* < 0) associations, respectively; asterisks (*) indicate a statistically significant correlation (*P* < 0.05). (For interpretation of the references to color in this figure legend, the reader is referred to the web version of this article.)

Partial least squares regression is a multivariate statistical technique used to analyze and predict linear relationships between variables, which boasts multivariate analysis, robust modeling, predictive accuracy, resilience to outliers, and visual interpretability (Yuan et al., 2022). We employed volatile compounds in ginkgo chicken soup as the independent variable (X) and non-volatile compounds as the dependent variable (Y). The partial least squares regression results are shown in Fig. 7, where a closer distance between X and Y indicates a stronger correlation between variables. The FAs C18:1n9t, C18:2n6, and C20:2, and the amino acid tyrosine showed proximity to 2-pentanol and (E)-2-heptenal, indicating a significant positive correlation among these compounds. Furthermore, C14:1 and C20:3n6 were close to 2-pentyl furan, and C16:0 was closely associated with 4-methyl-2-pentanone, suggesting positive correlations.

To further investigate the relationship between volatile and non-volatile components in ginkgo chicken soup, a comprehensive correlation analysis was performed. This analysis employed the Pearson correlation coefficient to assess the linkage between principal olfactory compounds and their olfactory characteristics. The Pearson correlation coefficient, a standard tool in statistical analysis, measures the degree of association between a pair of variables. It ranges from −1 to 1, with a score of 0 signifying no correlation. Scores below zero suggest an inverse relationship, whereas those above zero denote a direct relationship (Yuan et al., 2022). Furthermore, Fig. 7b illustrates that tyrosine had a notably positive association with (E)-2-heptenal (*P* < 0.05) but had a significant inverse relationship with both acetone and 3-methyl butanal (*P* < 0.05). C16:0 (palmitic acid) was inversely correlated with ethyl octanoate, 1-nonanal, and propyl acetate (*P* < 0.05). C18:0 (stearic acid) correlated positively with acetone, 3-methyl butanal, and 2-butanone (*P* < 0.05) and negatively with 2-pentanol and (E)-2-heptenal (*P* < 0.05). When FAs are heated, they decompose into various volatile compounds such as aldehydes, ketones, alcohols, and acids. SFAs such as palmitic acid (C16:0) and stearic acid (C18:0) primarily form aldehydes (e.g., 1-nonanal) and ketones (e.g., 2-butanone) through thermal oxidation and β-oxidation (Shakoor et al., 2022; Tanimoto et al., 2015). FAs such as palmitic acid (C16:0) and stearic acid (C18:0) demonstrated distinct relationships with various volatile compounds, highlighting their decomposition into aldehydes and ketones during heating. C18:1n9t (trans-oleic acid) showed a positive correlation with 2-pentanol (*P* < 0.05), and a negative correlation with acetone and 3-methyl butanal (*P* < 0.05). C18:2n6 (linoleic acid) was positively correlated with 2-pentanol (*P* < 0.05). C20:2 (eicosadienoic acid) was positively correlated with 2-pentanol and (E)-2-heptenal (*P* < 0.05) and negatively with acetone, 3-methyl butanal, and 2-butanone (*P* < 0.05). Linoleic acid (C18:2n6), an unsaturated FA, is prone to oxidation, resulting in the formation of aldehydes, such as (E)-2-heptenal, and alcohols, such as 2-pentanol (Tanimoto et al., 2015). Unsaturated FAs like linoleic acid (C18:2n6) were linked to the production of specific aldehydes and alcohols through oxidation processes.

## 4 Conclusions

This study compared the differences in flavor profiles of ginkgo chicken soup at different cooking times (30, 60, 90, 120, and 150 min) when prepared using a multifunctional combi oven and a traditional ceramic pot. The PCA results from the *E*-nose and E-tongue indicated that soups prepared using a combi oven and ceramic pot exhibited similar aroma and taste profiles. Our analysis of combi-cooked soup revealed 64 distinct volatile odorants, with 24 being characterized as pivotal contributors to the aroma, as determined by GC-IMS. Additionally, 17 amino acids and 6 nucleotides were identified, with the sweet and umami amino acid content increasing with increasing cooking time. The CO-5 sample contained higher levels of aldehydes, ketones, alcohols, and heterocyclic compounds than the CP sample prepared with a ceramic pot. According to TAV values, histidine, glutamic acid, and IMP were major contributors to the taste of ginkgo chicken soup. Furthermore, 18 FAs were identified, including 6 SFAs, 7 MUFAs, and 5 PUFAs. OPLS-DA revealed that 26 volatile and 11 non-volatile compounds contributed to flavor differences between soup samples. Sample CO-5 showed lower levels of TN, TFAA, umami and sweet amino acids, as well as MUFAs and PUFAs, compared to sample CP. Partial least squares regression modeling and Pearson's correlation identified significant relationships between FAs and aromatic compounds such as (E)-2-heptenal and 2-pentanol, which are derived from FA oxidation. Our research demonstrates that combi-oven-prepared ginkgo chicken soup matches traditional methods in flavor and aroma, endorsing its industrial application. Despite this, the study is limited by the absence of sensory evaluation, narrow chemical analysis, small sample size, and linear modeling. Future work should integrate human sensory data, examine flavor stability, broaden chemical scope, increase sample size, and consider non-linear models for enhanced predictive accuracy.

## CRediT authorship contribution statement

**Lilan Chen:** Writing – original draft, Investigation, Data curation. **Jiale Huang:** Writing – original draft. **Can Yuan:** Writing – review & editing, Methodology, Conceptualization. **Songcheng Zhan:** Conceptualization. **Mingfeng Qiao:** Methodology, Conceptualization. **Yuwen Yi:** Investigation, Formal analysis. **Chunyou Luo:** Software. **Ruixue Ma:** Data curation.

## Declaration of competing interest

The authors declare that they have no known competing financial interests or personal relationships that could have appeared to influence the work reported in this paper.

## Data Availability

Data will be made available on request.
